# Forward genetics screen coupled with whole-genome resequencing identifies novel gene targets for improving heterologous enzyme production in *Aspergillus niger*

**DOI:** 10.1007/s00253-017-8717-3

**Published:** 2018-01-06

**Authors:** Morgann C. Reilly, Joonhoon Kim, Jed Lynn, Blake A. Simmons, John M. Gladden, Jon K. Magnuson, Scott E. Baker

**Affiliations:** 1 Joint BioEnergy Institute, Emeryville, CA 94608 USA; 2 Chemical and Biological Processes Development Group, Pacific Northwest National Laboratory, Richland, WA 99352 USA; 3 Naval Medical Research Unit Dayton, Wright-Patterson Air Force Base, Dayton, OH 45433 USA; 4 Biological Systems and Engineering Division, Lawrence Berkeley National Laboratory, Berkeley, CA 94720 USA; 5 Biomass Science and Conversion Technologies Department, Sandia National Laboratories, Livermore, CA 94551 USA; 6 Biosystems Design and Simulation Group, Environmental Molecular Sciences Division, Pacific Northwest National Laboratory, Richland, WA 99352 USA

**Keywords:** *Aspergillus niger*, Heterologous protein expression, Forward mutagenesis, Whole-genome resequencing, MstC

## Abstract

Plant biomass, once reduced to its composite sugars, can be converted to fuel substitutes. One means of overcoming the recalcitrance of lignocellulose is pretreatment followed by enzymatic hydrolysis. However, currently available commercial enzyme cocktails are inhibited in the presence of residual pretreatment chemicals. Recent studies have identified a number of cellulolytic enzymes from bacteria that are tolerant to pretreatment chemicals such as ionic liquids. The challenge now is generation of these enzymes in copious amounts, an arena where fungal organisms such as *Aspergillus niger* have proven efficient. Fungal host strains still need to be engineered to increase production titers of heterologous protein over native enzymes, which has been a difficult task. Here, we developed a forward genetics screen coupled with whole-genome resequencing to identify specific lesions responsible for a protein hyper-production phenotype in *A. niger*. This strategy successfully identified novel targets, including a low-affinity glucose transporter, MstC, whose deletion significantly improved secretion of recombinant proteins driven by a glucoamylase promoter.

**Electronic supplementary material:**

The online version of this article (10.1007/s00253-017-8717-3) contains supplementary material, which is available to authorized users.

## Introduction

Biofuels and bio-based chemicals derived from lignocellulose are promising alternatives to traditional petroleum-originating fuels and chemicals. The plant cell wall is a complex assembly of cellulose, hemicellulose, lignin, pectin, and proteins (Somerville et al. 2004). Breaking down this heterogeneous substrate into its constituent monomers and oligomers requires a combination of physical, chemical, and/or biological deconstruction methods. A variety of chemical pretreatment methods have been explored, including acids, bases, solvents, and oxidation (Silveira et al. 2015). For the subsequent enzymatic hydrolysis steps to be both economically and environmentally viable, it is necessary to identify proteins that can remain active in the presence of residual pretreatment chemicals. It is also imperative that these enzymes can be produced economically and at a large scale.

Ionic liquid (IL) solvents show great promise as a method for biomass pretreatment (Yu et al. 2016). There has been a concerted effort to identify cellulose cocktail components that are IL-tolerant as current commercial mixtures are not. For example, a number of cellulolytic enzymes with thermophilic and IL-tolerant characteristics were identified from a switchgrass-degrading bacterial community (Gladden et al. 2011). However, many of the species producing these enzymes are not easily cultured. The predicted cellulolytic bacterial enzymes were produced either in vitro or from *Escherichia coli* in order to characterize their activity over a range of temperature, pH, and IL concentration (Gladden et al. 2014). In order to produce these enzymes at a larger scale, it was decided to clone these sequences into the fungal host, *Aspergillus niger* (Campen et al. 2017).

Filamentous fungi are commonly used for the commercial production of chemicals and enzymes. The ability of *A. niger* to grow aerobically over a wide range of both temperatures and pHs has repeatedly proved advantageous in industrial settings (Schuster et al. 2002; Oliveira et al. 2011). In addition, the organism is non-pathogenic and does not produce the mycotoxins that are common in some other related fungal species. It is therefore categorized as GRAS (generally recognized as safe) by the US Food and Drug Administration, meaning that substances generated using *A. niger* are considered harmless as food additives (Schuster et al. 2002; van Dijck et al. 2003). Because of its established use in industry, numerous large-scale fermentation protocols for *A. niger* exist. Finally, *A. niger* has a well-developed molecular toolbox, including various auxotrophic and antifungal resistance markers, a fully sequenced genome, and various transformation protocols, thus making it conducive to genetic manipulation.

We used a forward mutagenesis strategy to identify potential means of enhancing heterologous enzyme production in *A. niger*. A strain engineered to produce one of the IL-tolerant bacterial β-glucosidases (BGs) was subjected to chemical mutagenesis and the resulting strains screened for increased enzyme production. Subsequent whole-genome resequencing of a dozen hyper-production mutants found hundreds of genetic lesions, and bioinformatics analysis revealed several loci potentially associated with heterologous enzyme hyper-production, including those that appear to be specific to the heterologous protein expression construct used. One of the identified loci was annotated as a low-affinity glucose transporter, *mstC*. We show that the deletion of *mstC* improves the heterologous enzyme production driven by a glucoamylase promoter (P_*glaA*_) by fourfold.

## Materials and methods

### Reagents and strains

Unless otherwise indicated, all reagents were obtained from Sigma (St. Louis, MO USA). The *A. niger* strains used throughout this study are listed in Table 1. For general maintenance, *A. niger* strains were cultured on slants of Potato/Dextrose (PD) Agar and incubated at 30 °C for 3 days to allow for hyphal growth and spore formation.

**Table 1:** Strains used in this study

Name	Genotype	Source
WT	11414	ATCC
GFP	11414 P_*glaA*_*-GFP-*T_*trpC*_*/hphA*	Campen et al. 2017
A5IL97 1.1	11414 P_*glaA*_*-A5IL97-*T_*trpC*_*/hphA*	Campen et al. 2017
J03	11414 P_*glaA*_*-J03-*T_*trpC*_*/hphA*	Campen et al. 2017
J03 ∆*mstC*	11414 P_*glaA*_*-J03-*T_*trpC*_*/hphA* ∆*mstC*/*ptrA*	This study
11414 *pyrG*^*−*^	11414 *pyrG*^*−*^	Chiang et al. 2011
∆*glaA*/P_*glaA*_-A5	11414 *pyrG*^*−*^ Δ*kusA*/Af*pyrG* Δ*glaA*/P_*glaA*_*-A5IL97-*T_*trpC*_*/hphA*	Campen et al. 2017
∆*glaA*/P_*gpdA*_-A5	11414 *pyrG*^*−*^ Δ*kusA*/Af*pyrG* Δ*glaA*/P_*gpdA*_*-A5IL97-*T_*trpC*_*/hphA*	Campen et al. 2017
∆*glaA*/P_*ecm33*_-A5	11414 *pyrG*^*−*^ Δ*kusA*/Af*pyrG* Δ*glaA*/P_*ecm33*_*-A5IL97-*T_*trpC*_*/hphA*	Campen et al. 2017
∆*glaA*/P_*glaA*_-A5 ∆*mstC*	11414 *pyrG*^*−*^ Δ*kusA*/Af*pyrG* Δ*glaA*/P_*glaA*_*-A5IL97-*T_*trpC*_*/hphA* ∆*mstC*/*ptrA*	This study
∆*glaA*/P_*gpdA*_-A5 ∆*mstC*	11414 *pyrG*^*−*^ Δ*kusA*/Af*pyrG* Δ*glaA*/P_*gpdA*_*-A5IL97-*T_*trpC*_*/hphA* ∆*mstC*/*ptrA*	This study
∆*glaA*/P_*ecm33*_-A5 ∆*mstC*	11414 *pyrG*^*−*^ Δ*kusA*/Af*pyrG* Δ*glaA*/P_*ecm33*_*-A5IL97-*T_*trpC*_*/hphA* ∆*mstC*/*ptrA*	This study

The J03 strain was generated when 11414 (from the American Type Culture Collection; Manassas, VA USA) was transformed with vector containing a codon-optimized sequence for a BG from *Thermobaculum terrenum* (J03) (Gladden et al. 2014; NCBI GenBank Accession: KY014126), driven by the *A. niger* P_*glaA*_ and followed by the *Aspergillus nidulans* tryptophan biosynthesis terminator (T_*trpC*_) (the expression construct is detailed in (Campen et al. 2017)). A similar plasmid expressing the green fluorescent protein (GFP) in place of the J03 sequence was used to produce the strain GFP.

The construction of strains with a BG from *Thermotoga petrophila* (A5) (Park et al. 2012) under the control of various promotors is detailed in Campen et al. (2017). Briefly, the *Aspergillus fumigatus pyrG* (Af*pyrG*) was used to replace the native *kusA* locus in a 11414 *pyrG*^−^ strain (Chiang et al. 2011). This strain was subsequently transformed with a vector containing the P_*glaA*_-A5-T_*trpC*_ construct described above flanked by 1 kb of sequence 5′ and 3′ to the native *glaA* locus, allowing for targeted integration. In variations of this procedure, the A5 sequence was preceded by promoter sequences from either the *A. niger* glyceraldehyde-3-phosphate dehydrogenase or extracellular matrix loci (P_*gpdA*_ or P_*ecm33*_, respectively).

Generation of the *mstC* sugar transporter deletion strains—∆*mstC*, ∆*glaA*/P_*glaA*_-A5 ∆*mstC*, ∆*glaA*/P_*gpdA*_-A5 ∆*mstC*, and ∆*glaA*/P_*ecm33*_-A5 ∆*mstC* strains—is described below.

### Mutagenesis and primary plate screening

Spores were harvested from PD Agar slant tubes of J03 using a sterile solution of 0.02% (*v*/*v*) Tween-20. In a final volume of 0.5 mL, the spores were combined with 400 ng/mL 4-nitroquinoline 1-oxide (4-NQO; concentration empirically determined to result in a ~ 90% kill rate) and incubated on a shaker set at 200 rpm at 37 °C for 30 min. An equal volume of 5% (*v*/*v*) sodium thiosulfate was added to inactivate the 4-NQO before plating the spores on BG Screening Agar (20 g peptone, 8 g ammonium sulfate, 10 g ox-bile, and 15 g agar were added to 800 mL ddH_2_O and autoclaved; after autoclaving, 200 mL of a sterile 50% (*w*/*v*) maltose solution, 1 g esculin dissolved in methanol, and 5 mL of a sterile 10% (*w*/*v*) ferric citrate solution was added). The plates were incubated at 30 °C for 3 days before evaluating the esculin hydrolysis halos around the colonies in comparison with colonies from untreated J03 spores. Mutated colonies with increased halos compared to the parent were picked and transferred to slants of PD Agar.

### Secondary liquid culture screening

To evaluate the BG production of the *A. niger* mutant strains in liquid culture, 1 × 10^6^ spores/mL were first inoculated into 5 mL of CSL Medium (100 g corn steep liquor (50% (*w*/*v*) solids), 50 g fructose, 10 g glucose, 1 g sodium phosphate, 0.5 g magnesium sulfate, and 0.05 ml Antifoam 204 were added to 760 mL ddH_2_0, the pH adjusted to 5.8, and autoclaved; after autoclaving, 240 mL of a sterile 50% (*w*/*v*) maltose solution was added) in 60-mL glass culture tubes. The tubes were incubated in a shaker set at 200 rpm at 30 °C for 48 h to generate fungal biomass. An aliquot of 0.5 mL of culture was then transferred into 5 mL of HMM Medium (120 g maltose, 70 g sodium citrate (tribasic dihydrate), 15 g ammonium sulfate, 1 g sodium phosphate, 1 g magnesium sulfate (anhydrous), and 3 g SC Complete Media (Sunrise Science; San Diego, CA USA) were added to 1 L ddH_2_0, the pH adjusted to 6.2, and filter-sterilized) in 60-mL glass culture tubes. The tubes were incubated in a shaker set at 200 rpm at 30 °C for 120 h. Cell-free aliquots of the resulting culture supernatants were harvested using a 0.45 μm nylon centrifugal filter (VWR; Radnor, PA USA).

Aliquots from the culture supernatants were assayed for total secreted protein using the Bradford Protein Assay Kit (Bio-Rad; Hercules, CA USA). The BG activity in each sample was evaluated by combining 10 μL of culture supernatant with 90 μL of reaction mix (80 mM MES Buffer (pH 6.5), 5 mM 4-nitrophenyl-β-D-glucopyranoside (pNPG), plus or minus 10% (*v*/*v*) of the ionic liquid 1-ethyl-3-methylimidazolium acetate ([C_2_mim]OAc)). The reactions were incubated at 65 °C for 30 min before being quenched with 100 μL 2% (*w*/*v*) sodium carbonate. The absorbance of 100 μL aliquots of the pNPG reactions was read at 410 nm.

### Characterization of mutant strains

*A. niger* strains were first inoculated at 1 × 10^6^ spores/mL into 50 mL CSL Medium in 250-mL glass flasks and then sub-cultured at 5 mL into 50 mL HMM Medium in 250-mL glass flasks in triplicate for each strain. Cell-free samples of the resulting culture supernatants were harvested and assayed for total protein and BG activity as outlined above. In addition, the fungal biomass from each culture was harvested by decanting the culture through a single layer of Miracloth (EMD Millipore; Billerica, MA USA), pressing away the moisture, and then lyophilizing before weighing.

### Sequencing and bioinformatics analyses of mutants

Genomic DNA was prepared for sequencing by grinding ~ 0.5 g lyophilized fungal biomass to a fine powder and combining it with 15 mL CTAB Buffer (2% (*w*/*v*) hexadecyltrimethylammonium bromide, 100 mM Tris-HCl (pH 8), 20 mM EDTA (pH 8), and 1.4 M NaCl); the sample was vortexed to mix and then incubated at 57 °C for 2 h with occasional vortexing. An equal volume 25:24:1 phenol:chloroform:isoamyl alcohol was added to the sample, vortexed to mix, and then centrifuged at 4 °C for 10 min at 10,000 rcf. The resulting supernatant was extracted a second time with 5 mL 25:24:1 phenol:chloroform:isoamyl alcohol. Nucleic acids were precipitated by adding one volume 2-propanol to the resulting supernatant and centrifuging at 4 °C for 10 min at 4500 rcf. The resulting pellet was washed with 70% (*v*/*v*) ethanol, allowed to air-dry, and then incubated at 65 °C in 1 mL sterile ddH_2_O to resuspend. To remove RNA, 2 μL 200 mg/mL RNase A (Thermo Fisher Scientific; Waltham, MA USA) was added and the sample incubated at 37 °C for 2 h. The sample was extracted a final time with 1 mL 25:24:1 phenol:chloroform:isoamyl alcohol, and the DNA precipitated with the same 2-propanol and 70% (*v*/*v*) ethanol method as above before resuspending in 0.5 mL sterile ddH_2_O.

DNA libraries were produced at the Joint Genome Institute (JGI) and sequenced by the Illumina paired-end sequencing method using MiSeq 2 × 150 bp (~ 30× coverage) or HiSeq 2 × 100 bp (~ 100× coverage). The sequence data have been deposited with the NCBI BioProject database under the following accession numbers: PRJNA249619 (J03), PRJNA249388 (J03 1.1), PRJNA249608 (J03 1.2), PRJNA249607 (J03 1.6), PRJNA249612 (J03 1.7), PRJNA249480 (J03 1.10), PRJNA249613 (J03 2.8), PRJNA249614 (J03 4.3), PRJNA259122 (J03 6.3), PRJNA259125 (J03 7.2), PRJNA259124 (J03 7.4), PRJNA259128 (J03 8.2), and PRJNA259127 (J03 8.3). The sequenced reads were mapped to the reference genome sequence of *A. niger* strain ATCC 1015 v4.0 (http://genome.jgi.doe.gov/Aspni7/Aspni7.home.html), augmented with the sequence of the transformation plasmid containing J03, using BWA-MEM (Li 2014). Mapped reads were sorted by coordinate using SAMtools (Li et al. 2009), and duplicate reads were marked using Picard Tools (http://broadinstitute.github.io/picard).

Variant calling for single nucleotide polymorphisms and insertions/deletions was performed using a combination of BCFtools (Li 2011) or GATK tools (McKenna et al. 2010). Using BCFtools, variants calling and genotyping were done for each chromosome using multiple samples and merged. Using GATK, variants were called for each sample by GATK HaplotypeCaller, and joint genotyping of multiple samples was performed using GATK GenotypeGVCFs. Variants called by BCFtools were filtered using VCFtools, and variants called by GATK were filtered using GATK VariantFiltration. Filtered variants were annotated using the VariantAnnotation package in R (Obenchain et al. 2014). Structural variations such as insertion, deletion, or duplication of relatively large segments were identified using Pindel (Ye et al. 2009), BreakDancer (Chen et al. 2009), Delly (Rausch et al. 2012), or Lumpy (Layer et al. 2014).

### Deletion of sugar transporter

For targeted deletion of the MstC sugar transporter, a plasmid containing the *Aspergillus oryzae* pyrithiamine resistance (*ptrA*) sequence (Kubodera et al. 2000), in a reverse orientation and flanked by ~ 1 kb sequence upstream and downstream of the *mstC* locus, was synthesized (GenScript; Piscataway, NJ USA). The deletion construct was amplified by PCR and then transformed into the J03, ∆*glaA*/P_*glaA*_-A5, ∆*glaA*/P_*gpdA*_-A5, and ∆*glaA*/P_*ecm33*_-A5 strains similar to the protocol described by Yang et al. (2014). Briefly, 5 × 10^5^ spores/mL were inoculated into 100 mL yeast extract/peptone/dextrose (YPD) medium in a 250-mL glass flask and incubated in a shaker set at 150 rpm at 30 °C for 16 h. The culture was filtered through a single layer of sterilized Miracloth: mycelia retained on the Miracloth were thoroughly rinsed with sterile ddH_2_O and then transferred to a 250-mL glass flask containing 40 mL Protoplasting Buffer (0.6 M ammonium sulfate, 50 mM maleic acid, and 30 mg/mL VinoTaste Pro (Novozymes; Davis, CA USA) in ddH_2_O, the pH adjusted to 5.5, and filter-sterilized). The digesting mycelia were incubated in a shaker set at 70 rpm at 30 °C for 4–6 h. The culture was filtered through a single layer of sterilized Miracloth, and the resulting flow-through was centrifuged for 10 min at 800 rcf. The resulting pellet was washed twice with 25 mL ST Solution (1 M sorbitol in 50 mM Tris (pH 8.0), filter-sterilized) and once with 10 mL STC Solution (1 M sorbitol and 50 mM calcium chloride in 50 mM Tris (pH 8.0)), centrifuging for 10 min at 800 rcf. The protoplast pellet was resuspended in STC Solution to a concentration of 1.2 × 10^7^ protoplasts/mL and then combined with one-quarter volume PEG Solution (40% (*w*/*v*) polyethylene glycol 4000, 1 M sorbitol, and 50 mM calcium chloride in 50 mM Tris (pH 8.0), filter-sterilized); to this, dimethyl sulfoxide was added at 7% of the final volume. The purified deletion construct PCR product was added at 1–10 μg per 100 μL protoplast suspension and incubated on ice for 15 min; 1 mL PEG Solution was added and incubated at room temperature for 15 min. Next, 10 mL thiamine-minus sorbitol medium (10 g/L glucose, nitrate salts (6 g/L sodium nitrate, 0.52 g/L potassium chloride, 0.52 g/L magnesium sulfate heptahydrate, and 1.52 g/L potassium dihydrogen phosphate (Pontecorvo et al. 1953)), trace elements (2.25 mg/L zinc sulfate heptahydrate, 11 mg/L boric acid, 5 mg/L manganese chloride tetrahydrate, 5 mg/L iron sulfate heptahydrate, 1.7 mg/L cobalt chloride hexahydrate, 1.6 g/L copper sulfate pentahydrate, 0.085 mg/L ammonium molybdate dihydrate, and 50 mg/L tetrasodium ethylenediaminetetraacetic acid (Barratt et al. 1965)), thiamine-minus vitamin stock solution (1 mg/L each of biotin, nicotinic acid, *p*-aminobenzoic acid, pyridoxine, and riboflavin (Barratt et al. 1965)), and 1 M sorbitol in ddH_2_O, autoclaved to sterilize) was added and incubated in a shaker set at 80 rpm at 30 °C for 1 h. The sample was then centrifuged for 15 min at 800 rcf, and the resulting pellet resuspended in 12 mL pyrithiamine sorbitol agar (10 g/L glucose, nitrate salts, trace elements, thiamine-minus vitamin stock solution, 1 M sorbitol, 18 g/L agar, and 0.1 μg/mL pyrithiamine hydrobromide in ddH_2_O, autoclaved to sterilize) before plating; an overlay of 12 mL pyrithiamine sorbitol agar was applied, and the plate incubated at 30 °C for 3–5 days.

Pyrithiamine-resistant colonies were transferred to PD Agar slants. Spores were inoculated into 3 mL YPD Medium in 15-mL culture tubes and incubated in a shaker set at 200 rpm at 30 °C for 2–3 days. Fungal biomass was harvested using a 0.45-μm nylon centrifugal filter, and genomic DNA was prepared using the ZR fungal/bacterial DNA MiniPrep kit (Zymo Research; Irvine, CA USA). The strains were screened by PCR using primer pairs targeted within and flanking the ∆*mstC* construct to identify those strains where the *ptrA* sequence had replaced the *mstC* locus.

### Protein gel electrophoresis

Aliquots of cell-free culture supernatant prepared from CSL Medium and HMM Medium enzyme induction cultures of *A. niger* were combined with Laemmli sample buffer and heated to 99 °C for 10 min. Samples were then loaded on a 12% or 8–16% gradient Mini-Protean TGX Precast Gel (Bio-Rad; Hercules, CA USA) and run at a constant voltage. Coomassie staining was performed using GelCode Blue Stain Reagent (Thermo Fisher Scientific; Waltham, MA USA).

## Results

### Mutagenesis screen

**Fig. 1:**
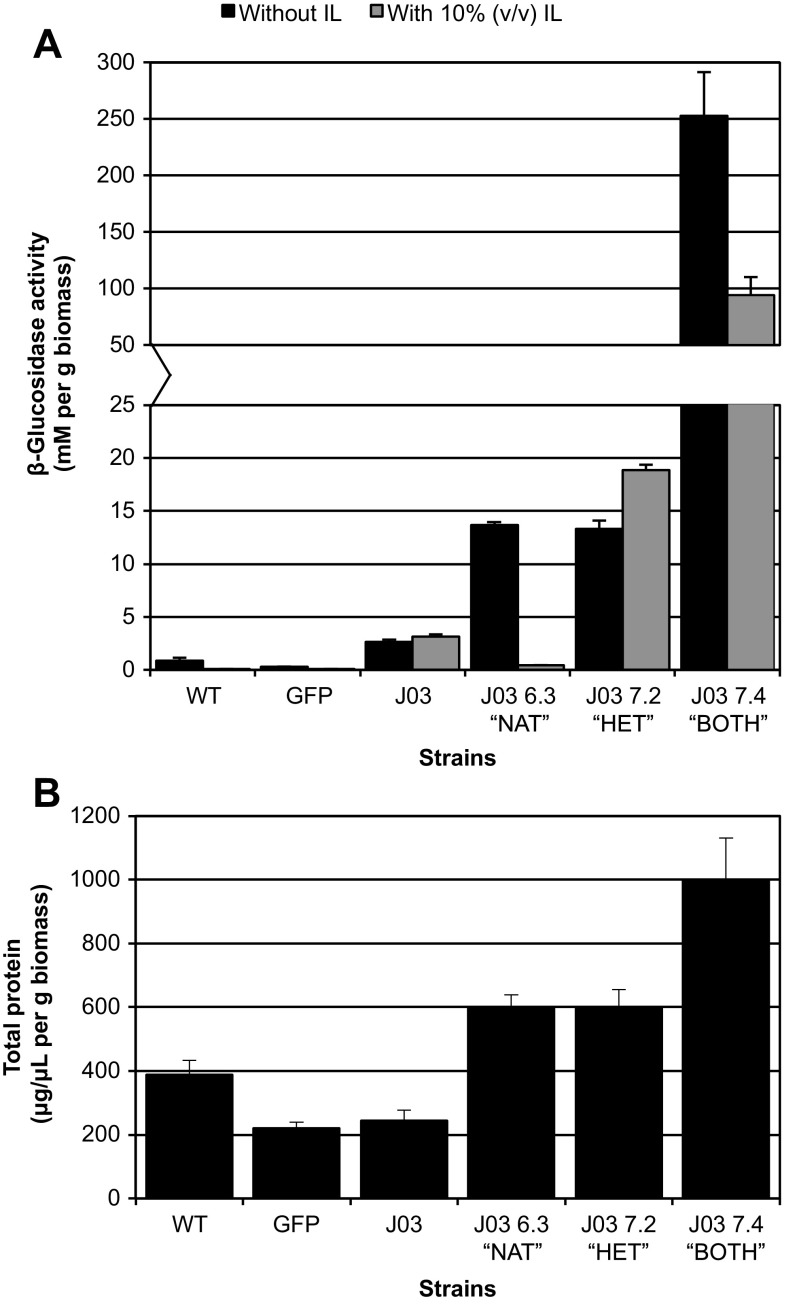
Growth of WT, GFP, J03, and J03-derivative strains in inducing culture conditions (CSL➔HMM). **a** β-glucosidase activity in culture supernatant as measured using the described pNPG assay: addition of ionic liquid [C_2_mim]OAc to reactions allows for distinction between native and heterologous enzymes. Enzyme activity (millimolar equivalents) was normalized to dry weight of fungal biomass (g). The addition of ionic liquid to the reactions allows for distinction between native and heterologous enzyme activity. **b** Total secreted protein as measured by Bradford assay (μg/μL) and normalized to dry weight of fungal biomass (g). Data drawn from biological replicates (*n* = 3); error bars indicate standard deviation

The *A. niger* strain ATCC 11414 (WT) was engineered to produce a BG from the bacterium *T. terrenum*, generating the strain J03. This enzyme was previously identified in a screen for thermophilic cellulose-degrading enzymes capable of functioning in the presence of the IL 1-ethyl-3-methylimidazolium acetate ([C2mim][OAc]) (Gladden et al. 2014). The fungal expression construct was designed using enzyme sequence codon-optimized to the genome of *A. niger*, driven by the *A. niger* P_*glaA*_, and followed by a terminator from one of the *A. nidulans* T_*trpC*_ (Campen et al. 2017). The *A. niger* J03 strain generated BG activity at moderately elevated levels compared to the parent (Fig. 1a) without significant change in the overall amount of protein secreted (Fig. 1b). It was possible to distinguish the activities of any native *A. niger* BG present in the culture supernatant samples from the heterologous bacterial enzyme through the inclusion of IL in the enzymatic assays: while the BG activity of both WT and a GFP control strain are reduced to background levels in the presence of IL, the BG activity associated with IL tolerant BG from *T. terrenum* (J03) in the J03 strain is unaffected (Supplemental Fig. S1B). A forward mutagenesis screen was designed using the J03 strain in an effort to identify loci that could enhance heterologous protein production.

Before carrying out the mutagenesis, a plate-based means of detecting secreted BG production was developed. First, a panel of colorimetric and fluorescent substrates was tested, including 5-bromo-4-chloro-3-indolyl β-D-glucopyranoside (X-Glc), esculin in combination with ferric citrate, indoxyl β-D-glucoside, 4-methylumbelliferyl β-D-glucopyranoside (MUGlc), and resorufin β-D-glucopyranoside. Culture supernatant from either J03 or the GFP control strain was spotted onto minimal media plates with these substrates infused into the agar: all but the indoxyl β-D-glucoside was found to allow for halo formation. However, when conidial spores from the same two strains were spread on the plates, only those containing X-Glc or esculin produced halos around the resulting colonies. Next, we found that a solid medium containing high levels of peptone and maltose produced the desired expression of recombinant BG from J03 while repressing the native BG secretion in the WT parent and GFP control strains. Finally, three colony-restricting compounds were tested: the detergent Triton X-100, the IL [C2mim][OAc], and ox-bile. Triton X-100 was effective in reducing colony size, but not at allowing for distinction between positive and negative (J03 and GFP, respectively) control strains while [C2mim][OAc] did not sufficiently restrict colony size, though it was effective at reducing native BG activity. Ox-bile (1% *w*/*v*) was chosen for the mutagenesis screen, as it was most effective at limiting colony size while still allowing for clear BG halo formation.

Once a means of screening for BG hyper-secretors was optimized, conidia from the J03 strain were mutagenized using 4-NQO to introduce point mutations in the genome and then spread onto plates with esculin, maltose, and ox-bile as detailed in the “Materials and methods” section. The spores were screened for large halo formation around colonies, and stocks were made of those colonies with dark halo diameters exceeding that of the J03 parent strain. These strains were subsequently tested for BG activity by growing the strain in first small (5 mL) and then large (50 mL) liquid cultures and testing the supernatant using the substrate pNPG to confirm the BG hyper-secretion phenotype.

Mutagenesis of conidia from J03 generated 12 mutant strains with consistently increased BG activity relative to the parent strain. These strains were categorized according to their ability to generate native and/or heterologous BG (Table 2). J03-derivative strains that maintained increased BG activity in the presence of 10% (*v*/*v*) [C2mim][OAc] were categorized as strains over-producing the heterologous J03 BG (HET), whereas native BG hyper-production mutants showed no IL-tolerance (NAT). Of the 12 mutants, 10 had increased J03 enzyme activity, while two were native BG hyper-producers (strains J03 4.3 and J03 6.3). Of note were those strains that showed reductions of up to ~ 50% BG activity in the presence of IL but did not reduce the activity to background as seen with the NAT strains. These strains—J03 1.2, J03 2.8, J03 7.4, and J03 8.3—were categorized as containing both elevated native and heterologous BG production (BOTH) relative to the parent. A representative of each phenotypic category can be seen in Fig. 1.

**Table 2:** Total protein production, β-glucosidase activity, and heterologous vs native enzyme production of J03-derivative strains

	Fold secreted total protein^a^	Fold secreted β-glucosidase activity^b^	Secreted β-glucosidase activity phenotype^c^
J03	1.0	1.0	–
J03 1.1	2.4	5.2	Heterologous
J03 1.2	3.7	63.3	Both
J03 1.6	2.2	4.6	Heterologous
J03 1.7	1.5	3.0	Heterologous
J03 1.10	1.0	2.0	Heterologous
J03 2.8	3.9	111.0	Both
J03 4.3	3.4	98.0	Native
J03 6.3	2.4	5.2	Native
J03 7.2	2.4	5.0	Heterologous
J03 7.4	4.1	95.8	Both
J03 8.2	2.4	4.3	Heterologous
J03 8.3	3.4	22.3	Both

^a^Secreted total protein was determined using Bradford assay and then normalized to dry weight of fungal biomass

^b^Secreted β-glucosidase activity was determined using a pNPG assay and then normalized to dry weight of fungal biomass

^c^Secreted β-glucosidase activity was evaluated in the absence or presence of 10% [C_2_mim]OAc. Changes of < 10% were considered to be primarily producing the J03 enzyme, the heterologous bacterial BG; changes of > 75% were primarily producing native BG of *A. niger*; changes of 30–60% were producing both heterologous and native BG

### Sequencing of hyper-production mutant strains

**Fig. 2:**
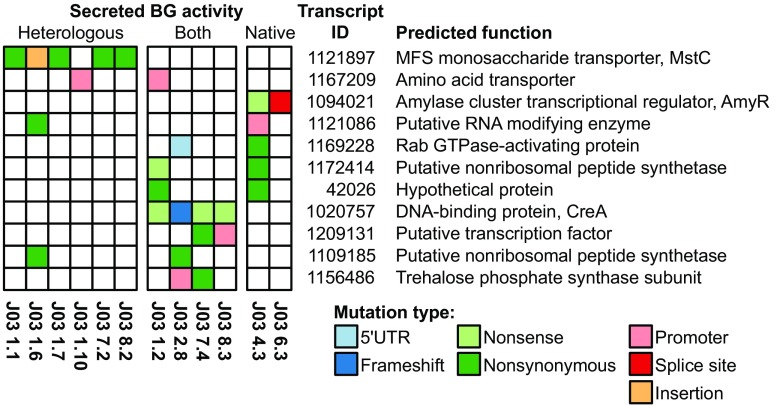
Mutations in J03-derivative strains associated with hyper-production of heterologous and/or native BG. Transcript ID references filtered the gene models of *A. niger* strain ATCC 1015 v4.0 (Aspni7 from the JGI); predicted function is based on nearest homolog or presence of conserved domains after BlastP analysis

To identify the genomic lesions responsible for the hyper-production phenotypes observed in the mutants, DNA was prepared from the 12 mutant J03-derivative strains as well as the parent strain J03 and WT. Genomic DNA was sequenced by the JGI and subsequently analyzed for mutations. Bcftools called 1727 variants and GATK called 1844 variants across all strains, and 1694 and 1326 variants passed fixed threshold filters, respectively. Among these, 962 variants called by both Bcftools and GATK were retained and subjected to annotation. Single nucleotide polymorphisms (SNPs) and small insertion/deletion (indel) calls were annotated using the filtered gene models of *A. niger* strain ATCC 1015 v4.0 (Aspni7 from the JGI). Variants in the coding regions, 5′ UTR, 3′ UTR, and splice sites (overlapping the first or last two nucleotides of an intron) were annotated first, followed by variants in the promoter region (500 bp upstream and 30 bp downstream of the transcription start site). Larger structural variants were visually inspected and manually annotated. Synonymous mutations in the coding region and variants present in the J03 parent strain were excluded from further analysis. The results of this analysis are presented in Fig. 2.

Of the six HET strains, five contained mutations (five missense and one insertion of ~ 9 kb sequence) within the coding region of Transcript ID 1121897, annotated as the low-affinity glucose transporter MstC (Supplemental Fig. S2). The one HET hyper-producer strains that does not have a mutation in *mstC*, J03 1.10, has a mutation in the promoter region of Transcript ID 1167209, a predicted amino acid transporter; this same promoter region was found to have a different mutation in one of the BOTH strains, J03 1.2.

All four of the BOTH strains have mutations (three independent nonsense and one frameshift) in Transcript ID 1020757, the DNA-binding carbon catabolite repression transcription factor CreA. Two of the BOTH strains (J03 7.4 and J03 8.3) have point mutations in either the coding or promoter region of Transcript ID 1209131, annotated as an unstudied fungal-specific transcription factor, while another pair (J03 2.8 and J03 7.4) have mutations in either the coding or promoter region of Transcript ID 1156486, a trehalose-6-phosphate synthase (TpsA), part of the trehalose synthesis pathway.

Finally, the two NAT strains have mutations in either the coding region or a splice site of Transcript ID 1094021, the amylase cluster transcriptional regulator AmyR. One of the NAT strains (J03 4.3) and one of the BOTH strains (J03 2.8) have mutations in either the 5′ UTR or coding region of Transcript ID 1169228, annotated as a Ypt/Rab GTPase-activating protein involved in intracellular vesicle transport. Interestingly, J03 4.3 and J03 2.8 are the two highest BG producers among the J03-derivative strains, with J03 4.3 far outperforming the other NAT strain, J03 6.3 (Table 2).

### Targeted deletion of mstC locus

**Fig. 3:**
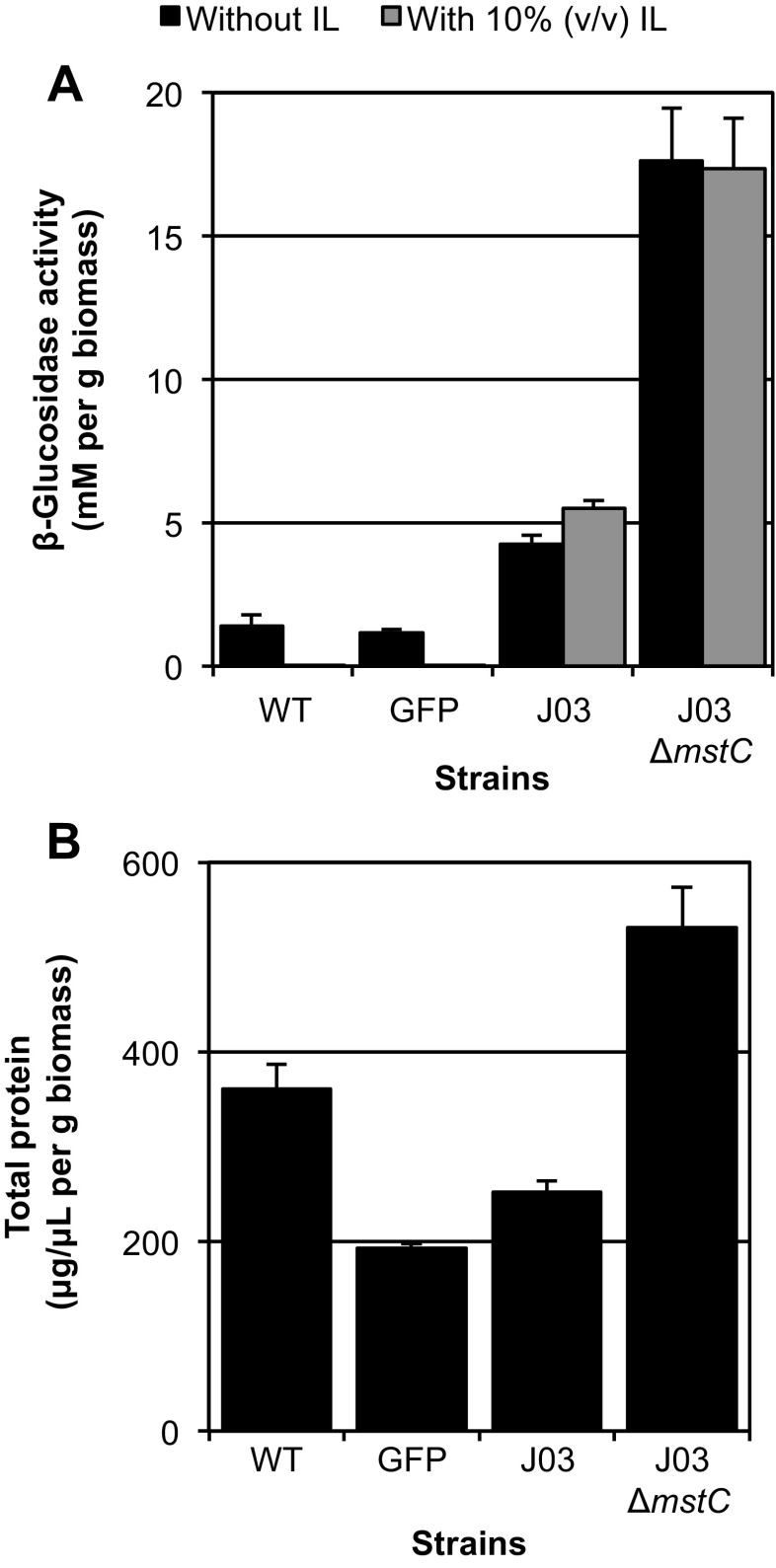
Loss of the *mstC* locus enhances heterologous enzyme production. **a** β-glucosidase activity and **b** total secreted protein of culture supernatant in CSL➔HMM inducing conditions (units as described in Fig. 1). Biological replicates *n* = 3; error bars indicate standard deviation

In order to establish an association between the identified loci and hyper-production of heterologous enzyme, deletion of the *mstC* locus was pursued. Initially, the *mstC* locus was targeted for deletion in the same J03 background that the mutagenesis screen had been performed in. Analysis of the BG activity of a J03 ∆*mstC* strain (Fig. 3) found elevated levels of BG compared to the parental J03 strain and that this activity persisted in the presence of IL, indicating it was the heterologous J03 protein as opposed to any native enzyme that was responsible for the increase in activity. As can be seen in Supplemental Fig. S3, proteins found in the culture supernatants of both wild-type *mstC* and ∆*mstC* strains yielded a similar banding pattern when examined by SDS-PAGE though the bands are more intense in the J03 ∆*mstC* strain, reflective of the total secreted protein levels for these samples (Fig. 3b).

**Fig. 4:**
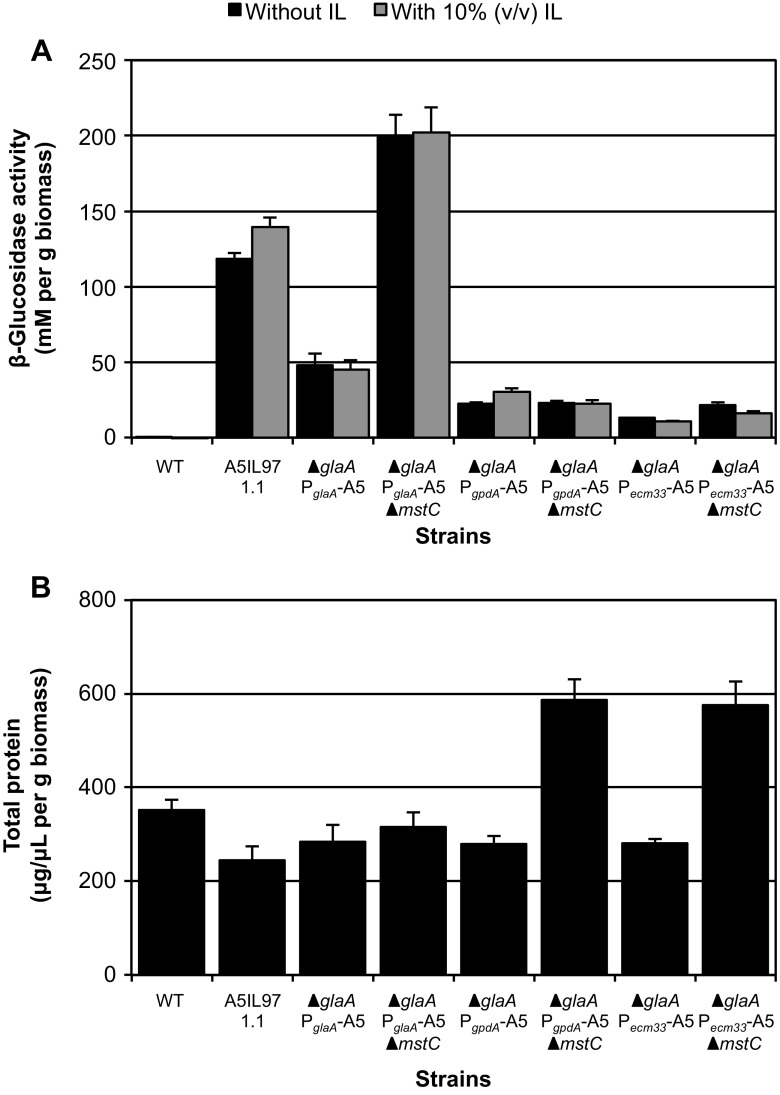
Impact of the *mstC* locus on enzyme production can be applied to other sequences in the heterologous expression construct but is limited to those behind the *glaA* promoter. **a** β-glucosidase activity and **b** total secreted protein of culture supernatant in CSL➔HMM inducing conditions (units as described in Fig. 1). Biological replicates *n* = 3; error bars indicate standard deviation. For clarity, relevant features of the strains—presence or absence of the native *glaA* and *mstC* loci, choice of promoter driving the heterologous β-glucosidase—are indicated at the bottom of the figure

We next explored whether the increased enzyme production in the absence of *mstC* was specific to the heterologous expression construct present in the original J03 strain. For this, strains in which the native *glaA* locus had been replaced by a bacterial BG from *Thermotoga petrophila*, A5 (Park et al. 2012), under control of one of several different promoters were utilized: ∆*glaA*/P_*glaA*_-A5, ∆*glaA*/P_*gpdA*_-A5, and ∆*glaA*/P_*ecm33*_-A5 (see “Materials and methods” section for description of assembly). These strains differ from the J03 ∆*mstC* strain in four respects: (1) expression of a distinct heterologous enzyme; (2) targeted as opposed to random integration of the expression construct; (3) deletion of the native *glaA* locus; and (4) use of additional constitutive promoters (P_*gpdA*_ and P_*ecm33*_) were tested. As can be seen in Fig. 4, the three different promoters allow for a small range of A5-associated BG activity (∆*glaA*/P_*glaA*_-A5 > ∆*glaA*/P_*gpdA*_-A5 > ∆*glaA*/P_*ecm33*_-A5). An additional strain expressing A5 (strain A5IL97 1.1) using the same construct as the original J03 was also utilized for this comparison. Deletion of the native *mstC* locus in these backgrounds clearly resulted in a fourfold increase in heterologous BG activity in the ∆*glaA*/P_*glaA*_-A5 strain but negligible differences in the ∆*glaA*/P_*gpdA*_-A5 and ∆*glaA*/P_*ecm33*_-A5 backgrounds. Interestingly, both the ∆*glaA*/P_*gpdA*_-A5 ∆*mstC* and ∆*glaA*/P_*ecm33*_-A5 ∆*mstC* strains show significantly increased total protein secretion compared to ∆*glaA*/P_*glaA*_-A5 ∆*mstC* (see Fig. 4B and Supplemental Fig. S4) even though any change in A5 expression compared to the parent strain is minimal. Although the impact of ∆*mstC* on protein production is apparently restricted to those heterologous loci placed behind the *glaA* promoter, it does not seem to be limited to a particular protein sequence as both the J03 and A5 enzyme activities could be elevated.

## Discussion

*A. niger* is frequently used for the industrial production of chemicals and proteins (Meyer et al. 2015). In the above study, we sought to identify means for enhancing the production of secreted heterologous proteins in this fungal host. To this end, we mutagenized a strain of *A. niger* engineered to secrete a bacterial BG enzyme (J03) and used an agar plate-based screen to identify isolates with improved BG activity compared to the parent strain. Mutant strains that continued to demonstrate consistently elevated enzyme activity in liquid culture were analyzed to distinguish between those strains producing the desired heterologous BG (HET strains) and those that were over-producing a native BG (NAT strains) (Table 2). Unexpectedly, this also led to the identification of a third phenotype: strains that were successfully hyper-secreting both the heterologous and native BG enzymes (BOTH strains).

The genome sequence of 12 mutant strains—six HET, four BOTH, two NAT—were compared to the parent J03 strain as well as the WT strain. The most common mutations in each phenotypic category were MstC (a low-affinity glucose transporter) for the HET strains, CreA (a transcription factor with roles in carbon catabolite repression) for the BOTH strains, and AmyR (a transcription factor that regulates the amylase cluster) for the NAT strains (Fig. 2). Sequence analysis also identified several other loci with possible ties to protein hyper-production that are shared between the phenotypic categories. The results are interesting both for those affected loci that are shared across phenotype categories and those that are unique.

Here, we have demonstrated that the absence of MstC clearly results in increased production of proteins behind the *glaA* promoter (Figs. 3 and 4). A member of the major facilitator superfamily, MstC is thought to be a low-affinity glucose transporter (Jorgensen et al. 2007). Transcriptional evidence initially indicated expression of *mstC* was associated with higher glucose concentrations in the culture medium (Jorgensen et al. 2007), but more recent studies showed that protein levels of MstC are relatively abundant and independent of the glucose concentration (Sloothaak et al. 2015). A mutant strain of *Saccharomyces cerevisiae* deficient in sugar transport can be complemented with MstC to support growth on fructose, galactose, glucose, and mannose (de Vries et al. 2017). It is well-established that the *glaA* promoter is induced by maltose, glucose, and other starch-related compounds (Fowler et al. 1990; Ganzlin and Rinas 2008). That mutation of *mstC* results in increased expression of genes under P_*glaA*_ could indicate that MstC is involved in nutrient signaling as a transceptor. Initially characterized in *Saccharomyces*, transceptors have amino acid similarity to—and may function as—transporters, but also act as signal-transducing receptors involved in sensing nutrient availability (Diallinas 2017; dos Reis et al. 2017; Ozcan et al. 1996). One possible explanation for the impact of ∆*mstC* on P*glaA*-controlled proteins is that *mstC* encodes (or its deletion induces) a transceptor involved in signaling levels of maltose, glucose, or a combination of the two. When the potential signaling pathway is disrupted, the fungus senses an incorrect maltose or glucose level that may induce rather than repress the secretion of glucoamylase and potentially other secreted enzymes.

It is accepted that CreA regulates BG activity in aspergilli (Lee et al. 1996) and that inhibition of CreA results in the secretion of native cellulolytic enzymes on non-inducing sugars (Ruijter and Visser 1997). Thus, it is not surprising that all four J03-derived BOTH strains have mutations in CreA (Table 2) and increased native BG activity. Inhibition of CreA is not known to elevate expression of *glaA* through its native promoter (Nakamura et al. 2006) and therefore would not be expected to regulate the P_*glaA*_-driven heterologous BG constructs used here. However, during characterization of the *A. nidulans* ortholog of MstC (*An*MstE), expression of this low-affinity glucose transporter was significantly reduced in CreA-depressed mutants grown in the presence of repressing carbon sources (Forment et al. 2006). If the *creA* mutations in the BOTH strains are causing a decrease in MstC, this may explain the increased levels of heterologous BG activity seen in these strains. This provides an interesting connection between the most commonly impacted loci in the J03-derivative HET and BOTH strains (*mstC* and *creA*, respectively).

The NAT-categorized strains, J03 4.3 and 6.3, were each found to have mutations in the *amyR* locus. AmyR is known to regulate production of amylolytic enzymes (Kowalczyk et al. 2014) but has also been linked to the production of native BG in *A. niger*. Disruption of *amyR* was shown to reduce the activity of BG, while overexpression of *amyR* led to increased BG activity (van Kuyk et al. 2012). Given that the two NAT strains exhibited elevated native BG activity compared to the parent strain, the mutations associated with *amyR* in these strains may enhance its expression or activity. The high BG activity of NAT strain J03 4.3 is more similar to that of the four BOTH strains than the other NAT strain (Table 2). J03 4.3 and the BOTH strain J03 2.8 each had a mutation in a locus identified as a Ypt/Rab GTPase-activating protein. Proteins in this family are typically involved in intracellular vesicle trafficking (Lipatova et al. 2015). It is possible that mutations in this locus in J03 2.8 and 4.3 resulted in increased secretory capabilities of the strains, whether it was the native or heterologous BG.

In the future, it would be valuable to identify the sugar that is being transported and/or sensed by MstC and thus responsible for providing the native suppressive effect of this protein. It would also be worthwhile to investigate some of the loci that were shared between different phenotypic categories. For example, the only HET strain that did not have identifiable mutations in the *mstC* locus, J03 1.10, has a mutation in a possible amino acid transporter; this same locus was also found to have a mutation in the BOTH strain J03 1.2. It would be interesting to see what impact the deletion or overexpression of this locus has on heterologous enzyme production in *A. niger*. Similarly, mutations in the Ypt/Rab GTPase-activating protein found in BOTH strain J03 2.8 and NAT strain J03 4.3 might further increase production of heterologous BG if introduced into one HET strain backgrounds. Together, these studies would add further to our understanding of heterologous protein production in *A. niger*.

## Electronic supplementary material

ESM 1 (PDF 1253 kb)
