# Supplementary material for: Forward genetics screen coupled with whole-genome resequencing identifies novel gene targets for improving heterologous enzyme production in Aspergillus niger
Source: Appl Microbiol Biotechnol. 2018 Jan 6;102(4):1797–807. doi: 10.1007/s00253-017-8717-3 (PMC5794824; doi:10.1007/s00253-017-8717-3)
Supplement: Supplementary file 1 — (PDF 1253 kb) [file 253_2017_8717_MOESM1_ESM.pdf]

JOURNAL: Applied Microbiology and Biotechnology

TITLE: Forward genetics screen coupled with whole-genome resequencing identifies novel gene targets for improving heterologous enzyme production in *Aspergillus niger*

AUTHORS: Morgann C. Reilly[1,2], Joonhoon Kim[1,2], Jed Lynn[1,3], Blake A. Simmons[1,4], John M. Gladden[1,5], Jon K. Magnuson[1,2], Scott E. Baker[1,6]

AUTHOR AFFILIATIONS:
[1] Joint BioEnergy Institute, Emeryville, CA, 94608, USA
[2] Chemical and Biological Processes Development Group, Pacific Northwest National Laboratory, Richland, WA, 99352, USA
[3] Naval Medical Research Unit Dayton, Wright-Patterson Air Force Base, OH, 45433, USA
[4] Biological Systems and Engineering Division, Lawrence Berkeley National Laboratory, Berkeley, CA, 94720, USA
[5] Biomass Science and Conversion Technologies Department, Sandia National Laboratories, Livermore, CA, 94551, USA
[6] Biosystems Design and Simulation Group, Environmental Molecular Sciences Division, Pacific Northwest National Laboratory, Richland, WA, 99352, USA

CORRESPONDING AUTHOR:
Scott E. Baker: scott.baker@pnnl.gov, 509-372-4759 (telephone)

SUPPLEMENTARY MATERIAL

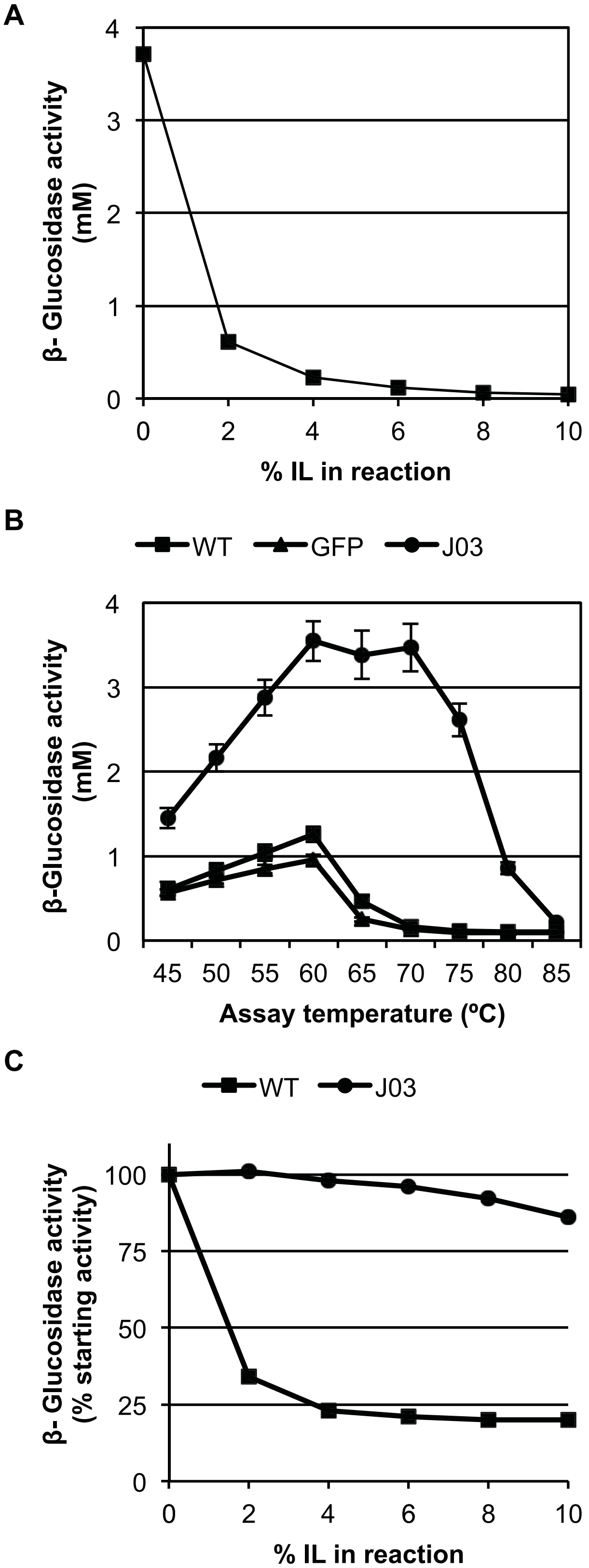

Fig. S1 β-Glucosidase activity assays. **(A)** β-Glucosidase activity of commercial *A. niger* glucosidase (Sigma 49291, 0.31 mg/mL). **(B)** β-Glucosidase activity in culture supernatants evaluated across temperature range. **(C)** β-Glucosidase activity at 65 °C determined across ionic liquid [C_2mim]OAc range.

A *tttctctcccatcaatcttctcttttcccatcttcccatccctggcagaggattccgcgttgtatgaatccaccggcaac*ATGGGTGTCTCTAATATGA
TGTCCCGGTTCAAGCCTCAGGCGGACCACTCTGAGTCCTCCACTGAGGCTCCTACTCCTGCTCGCTCCAACTCCGCCGTCGAGAAGGACAATGTCTTGCT
CGATGACAGTCCCGTCAAGTACTTGACCTGGCGCTCCTTCATCCTGGGTATCGTCGTGTCCATGGGTGGTTTCATCTTCG**C**TTACTCTACTGgtatggtg
acatcgattctctgcagctagtcccctcggttgctaaccttttccaccaccagGTCAAATCTCTGGTTTCGAGACTATGGATGACTTCCTCCAACGTTTC
GGTCAGGAACAGGCGGATGGATCCTATGCTTTCAGCAAC**A**TCCGTAGT**C**GTCTCATTGTCGGTCTGgtaagtggcatacatcatggactgtccctagaga
ccaacccgactgacaatctcttcagCTGTGTATCGGTACTATGATCGGTGCCCTGGTTGCTGCTCCTATCGCAGACCGCATGGGCCGCAAGCTCTCCATC
TGTCTCTGGTCTGTCATCCACATCGTCGGTATCATCATTCAGATTGCCACCGACTCCAACTGGGTCCAGGTCGCTATGGGTCGTTGGGTTGCCGGTCTGG
GTGTTGGTGCCCTCTCCAGCATTGTCCCCATGTACCAGAGTGAATCTGCTCCCCGTCAGGTCCGTGGTGCCATGGTCAGTGCCTTCCAGCTGTTCGTTGC
CTTCGGTATCTTCATCTCCTACATCATCAACTTCGGTACCGAGAGAATCCAGTCGACTGCTTCCTGGCGTATCACCATGGGCATTGGCTTCGCCTGGCCC
TTGATTCTGGCTGTTGGCTCTCTCTTCCTGCCCGAGTCTCCTCGTTTCGCCTACCGTCAGGGTCGTATCGATGAGGCCCGTGAGGTTATGTGCAAGCTGT
ACGGTGTCAGCCCGAACCACCGCGTCATCGCCCAG**A**AGATGAAGGACATGAAGGACAAGCTCGACGAGGAGAAGGCCGCCGGTCAGGCTGCCTGGCACGA
GCTGTTCACCGGCCCTCGCATGCTCTACCGTACCCTGCTCGGTATTGCTCTGCAGTCCCTCCAGCAGCTGACC**T**GTGCCAACTTTATCTTCTACTACGGA
AACAGTATCTTCACCTCCACTGGTCTGAGCAACAGCTACGTCACTCAGATCATTCTGGGTGCTGTCAACTTCGGTATGACCCTGCCCGGTCTGTACGTCG
TCGAGCACTTCGGTCGTCGTAACAGTCTGATGGTTGGTGCTGCCTGGATGTTCATTTGCTTCATGATCTGGGCTTCCGTTGGTCACTTCGCTCTGGATCT
TGCCGACCCTCAGGCCACTCCTGCCGCTGGTAAGGCCATGATCATCTTCACTTGCTTCTTCATTGTCGGTTTCGCCACCACCTGGGGTCCTATCGTCTGG
GCCATCTGTGGTGAGATGTACCCCGCCCGCTACCGTGCTCTCTGCATTGGTATTGCCACCGCTGCCAACTGGACCTGGAACTTCCTCATCTCCTTCTTCA
CCCCCTTCATCTCTAGCTCCATTGACTTCGCCTACGGCTACGTCTTTGCTGGATGCTGTTTCGCCGCCATCTTCGTTGTCTTCTTCTTCGTCAATGAGAC
CCAGGGTCGCACTCTTGAGGAGGTTGACACCATGTACGTGCTCCACGTCAAGCCCTGGCAGAGTGCCAGCTGGGTTCCCCCGGAGGGCATTGTCCAGGAC
ATGCACCGCCCCCCTTCCTCTTCCAAGCAGGAGGGTCAGGCTGAGATGGCTGAGCACACCGAGCCCACTGAGCTCCGCGAGTAA*gccactcgcactcgcg*
cgaactcattttgctagttgctcttgtacattgaacctgcatcttaagctttgatttatttaattgcatgattgctctttgcatcgcatttgctagctag
ctattatcggcatgaatgcgtccacgcacgcaatgtttgaatggcttgactcatcgggaaggatatggttgggaactacgacatcggcgtttggtgatac
ctgcaactgcatacatctgttgacgttgaaattttttttgaggttattgagaaacgatttaatacaagtatatacgaaccttacttggtg

B MGVSNMMSRFKPQADHSESSTEAPTPARSNSAVEKDNVLLDDSPVKYLTWRSFILGIVVSMGGFIF**G**YSTGQISGFETMDDFLQRFGQEQADGSYAFSN**V**
A **I**

RS**G**LIVGLLCIGTMIGALVAAPIADRMGRKLSICLWSVIHIVGIIIQIATDSNWVQVAMGRWVAGLGVGALSSIVPMYQSESAPRQVRGAMVSAFQLFVA
R

FGIFISYIINFGTERIQSTASWRITMGIGFAWPLILAVGSLFLPESPRFAYRQGRIDEAREVMCKLYGVSPNHRVIAQ**E**MKDMKDKLDEEKAAGQAAWHE
K

LFTGPRMLYRTLLGIALQSLQQLT**G**ANFIFYYGNSIFTSTGLSNSYVTQIILGAVNFGMTLPGLYVVEHFGRRNSLMVGAAWMFICFMIWASVGHFALDL
C

ADPQATPAAGKAMIIFTCFFIVGFATTWGPIVWAICGEMYPARYRALCIGIATAANWTWNFLISFFTPFISSSIDFAYGYVFAGCCFAAIFVVFFFVNET

QGRTLEEVDTMYVLHVKPWQSASWVPPEGIVQDMHRPPSSSKQEGQAEMAEHTEPTELRE*

Fig. S2 The *mstC* locus and encoded protein. **(A)** Native genomic DNA: italicized lowercase text is 5' UTR 3' UTR sequence, lowercase text is intron sequence, and uppercase text is exon sequence. Mutations in the J03-derivative strains J03 1.1, J03 1.6, J03 1.7, J03 7.2, and J03 8.2, are indicated either by bold red text for a nucleotide point mutation from native guanine or underlined text indicating an insertion site between two bases. **(B)** Native amino acid sequence: mutations in the strains J03 1.1, J03 1.6, J03 1.7, J03 7.2, and J03 8.2, are indicated either by bold red text for an amino acid change resulting from nucleotide change or by underlined text for an insertion site between two amino acid residues.

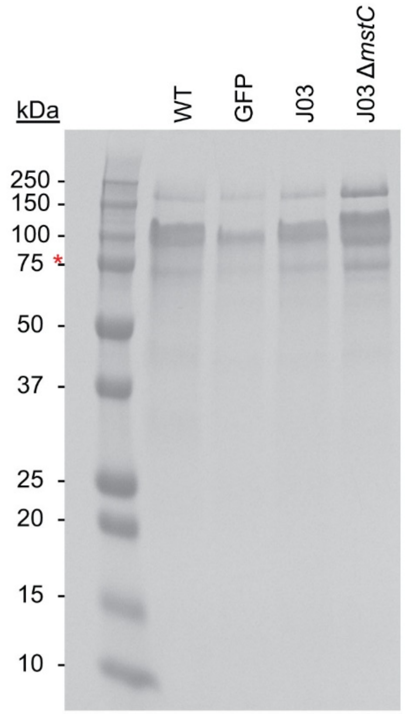

Fig. S3 Equal volumes of supernatant from CSL→HMM inducing cultures were loaded and separated by electrophoresis on 8-16% gradient polyacrylamide gels. Predicted molecular weight of J03 (~80 kDa) indicated with red *.

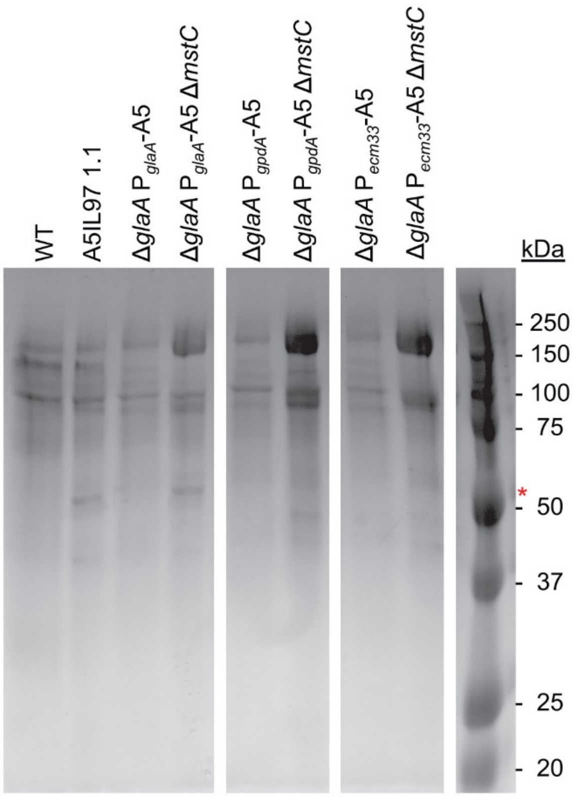

Fig. S4 Equal volumes of supernatant from CSL→HMM inducing cultures were loaded and separated by electrophoresis on 12% polyacrylamide gels. Predicted molecular weight of A5IL97 (~52 kDa) indicated with red *.
